# Polyacrylamide Grafted Xanthan: Microwave-Assisted Synthesis and Rheological Behavior for Polymer Flooding

**DOI:** 10.3390/polym13091484

**Published:** 2021-05-05

**Authors:** Souheyla Chami, Nicolas Joly, Patrizia Bocchetta, Patrick Martin, Djamel Aliouche

**Affiliations:** 1 Laboratory of Polymers Treatment & Forming, Faculty of Technology, M’Hamed Bougara University, Boumerdes 35000, Algeria; chamis2007@yahoo.fr (S.C.); aliouche.djamel@univ-boumerdes.dz (D.A.); 2 Univ Artois, UniLaSalle, Unité Transformations & Agroressources, ULR7519, F-62408 Béthune, France; nicolas.joly@univ-artois.fr; 3 Department of Innovation Engineering, University of Salento, Via per Monteroni, 73100 Lecce, Italy; patrizia.bocchetta@unisalento.it

**Keywords:** xanthan, polyacrylamide, rheology, viscosity loss, viscoelasticity, enhanced oil recovery

## Abstract

Application of polymer-flooding systems in secondary and tertiary oil recovery represents a real challenge for oil industry. In this work, our main objective is to explore possibilities of making use of xanthan-*g*-polyacrylamide for polymer flooding in a particular Devonian oilfield of medium salinity. The graft polymer was synthesized by using microwave-assisted graft copolymerization reaction of acrylamide on xanthan. The synthesized copolymer with optimized grafting parameters has been characterized by Infrared Spectroscopy and Thermal Analysis (DSC). Rheological analysis by steady shear and oscillatory flow experiments have been subsequently performed for xanthan and grafted xanthan under reservoir conditions. In steady shear, as expected the grafted polymer solutions flow as shear-thinning materials and apparent viscosity showed good fits with Cross’s model. The viscosity losses due to salinity or temperature are more controlled for the grafted xanthan compared to pristine xanthan. When the grafted polymer concentration is increased to 2000 ppm the losses were halved. In oscillatory shear, the copolymer solutions followed a global behavior of semi-dilute entangled systems; furthermore, all dynamic properties were influenced by the brine salinity. Compared to xanthan, the elastic properties of xanthan-g-polyacrylamide solutions have been significantly improved in saline media and the losses in elasticity of grafted polymer solutions are lower.

## 1. Introduction

In oilfield operations, some techniques are used to improve recovery of additional quantities of oil and gas from the depositional field. Suitability of a process for a particular oilfield is governed by the fluid properties and field environment geology [1]. Application of polymer flooding in Enhanced Oil Recovery (EOR) systems has become an efficient and cost-effective procedure [2]. According to wellbore conditions, the mobility of water and oil as well as the relative permeability in the core reservoir are complex parameters for recovering residual oil [3,4]. Polymer solutions are added with injected brine to increase its viscosity and, therefore, control the oil mobility and lower the core permeability, even at very low concentrations [5]. In order to upgrade sweep productivity in oil recovery techniques, the role of polymer solution will be to adjust viscosity of the aqueous phase and to behave as shear-thinning material. Shear-thinning solutions can delay flow disruption at low shear rates, but they do not resist enough to flow at high shear rates. For drilling fluids, the flow performance of polymer solution is fundamental, as during flooding in porous reservoirs, polymer rheology affects both injectivity and sweep efficiency. The polymer selected for EOR should have relevant properties for flooding: water-soluble, shear thickener for water; shear thinner for crude oil mixture, low coast and non-toxic [6,7]. Partially hydrolyzed polyacrylamide (HPAM) and polysaccharides (xanthan and cellulose products) were widely applied in polymer flooding procedures and extensively reviewed for their application in EOR management [8,9,10,11]. Compared to polysaccharides, with its low cost, HPAM polymer can yield solutions with desired viscosity levels even at very low concentrations [12]. However, due to the screening effect of charges carried by the acrylate groups on the polymer chains, flow behavior of polyacrylamide is more affected by the presence of salts. Therefore, in more salt aqueous phase the shear resistance of polyacrylamide polymers is poor and restricts its exploitation in high salinity reservoirs [13]. Xanthan, a second water-soluble polysaccharide applied for flooding, is less affected by the presence of salts and exhibits better rheological behavior than that of HPAM [14,15]. However, the aqueous xanthan solution is deficient in elasticity; additionally, use of xanthan is restricted in high temperature reservoirs due to its weak thermal stability [16]. To improve rheology of these polymers, several alternatives have been proposed, such as chemical modification of HPAM [17,18,19] or combination of xanthan with other polysaccharides [20,21,22,23]. In this context, it seems interesting to combine functional characteristics of both polymers by grafting polyacrylamide onto xanthan. In a previous work, polymer flooding using xanthan biopolymer was carried out to explore its potential for improving oil recovery in a particular Algerian oil field [24]. In the present study, we focus on the polymer flooding characteristics of xanthan-graft-polyacrylamide under the reservoir conditions. Behavior of both polymers was compared in terms of viscosity and viscoelastic properties. Xanthan-g-polyacrylamide was synthesized by a fast graft copolymerization reaction under microwave irradiation. To yield copolymer with optimal characteristics, we have adjusted some parameters of the grafting reaction: presence or absence of chemical initiator, irradiation power, reaction time, xanthan polymer, and acrylamide monomer amounts. The grafted xanthan so prepared was characterized by FTIR spectroscopy. Subsequently, xanthan and grafted xanthan were evaluated for polymer flooding through their behavior in deionized water (DW) and reservoir formation water (RW) of Devonian nature, a third brine of Liassic nature was used as injection water (IW). The rheological experiments for steady shear and dynamic oscillatory flow were completed for different polymer concentrations and temperatures and polymer solutions were prepared with the brines cited above, as solvents.

## 2. Materials and Methods

### 2.1. Materials

Xanthan polymer (Mw~5 × 10^6^ Da) was provided by AVA Drilling Fluids and Services Co., (Roma, Italy). For each experiment, polymer solution was prepared at constant temperature by slow addition of known amounts of gum in a reactor containing DW, IW or RW. Acrylamide monomer (AAm), from Sigma-Aldrich Chemie Gmbh (Munich, Germany), was purified by double recrystallization from methanol, vacuum dried at 40 °C, and then stored in a desiccator over silica gel. Initiator, Ammonium persulfate (APS), acetone, and methanol were all supplied by Panreac Monplet and Esteban, S.A. (Barcelona, Spain) and used without additional purification.

Deionized water was employed in the grafting reaction and preparation of polymer-DW solutions. In most oilfields, injection water and reservoir formation brine (both contain salts) are essential water sources; their impact on rheology of polymer solutions is very significant for the flooding. The brine composition has a considerable influence on the aqueous phase viscosity. In accordance with the field geology, two brines have been used for polymer mixing: Liassic brine as injection water, Devonian one as reservoir water. Table 1 shows the chemical composition of these brines. The internal pressure in the well is of 131.1 bars; the well internal temperature is about 68 °C.

### 2.2. Graft-Copolymerization of AAm onto Xanthan

The reaction synthesis of xanthan-g-polyacrylamide was carried out following the method described by Kumar et al. [25]. In this work microwave irradiation (MW) was exploited (in the presence and absence of APS initiator) to produce free radical sites on the polymer surface. Briefly, the desired amount of xanthan was dispersed in deionized water and stirred overnight. To this solution, a known amount of acrylamide was added, and the mixture was stirred until complete dissolution; for the grafting in the presence of redox initiator, a known amount of APS was included to this above solution. The so prepared solution was irradiated in a domestic microwave oven (Philips Whirlpool Model AVM 581), by varying the procedure parameters (time, power, concentration of AAm and xanthan amount) in order to prepare a range of grafted xanthan samples. After reaction, the grafted samples were washed first with acetone, then with a mixture of methanol-water to extract any unreacted monomer residues. Solid precipitates obtained were submitted to filtration and drying overnight under vacuum at 40 °C, then ground into powder and finally weighed up and kept steady in a desiccator. The grafting ratio (*G* %), grafting efficiency (*GE* %) and homopolymer fraction (*H* %) were calculated using the following equations:

(1) $$\left(\%G\right)=\left(\frac{W_{1}-W_{0}}{W_{0}}\right).100$$

(2) $$\left(\%GE\right)=\left(\frac{W_{1}-W_{0}}{W_{2}}\right).100$$

(3) (%H)=100−(%GE)

where *W_0_* is the weight of xanthan, *W_1_* the weight of graft copolymer, and *W_2_* the weight of acrylamide.

### 2.3. Solubility Tests

The main condition for using a polymer in EOR is the water-solubility. Solubility of grafted copolymers was tested in three different fluids: deionized water, injection, and formation brines. Suitable amounts of polymer powder were dissolved in the corresponding medium by stirring at room temperature. From the solubility test results, it was observed that all grafted samples were dissolved in a few minutes (3–5 min) in the three fluids. All graft copolymers were easily and completely soluble. After three weeks of storage in de-aerated conditions, they were still stable.

### 2.4. Preparation of Polymer Solutions

Xanthan and xanthan-g-polyacrylamide solutions at 500, 1000 and 2000 ppm concentrations were prepared in DW, IW and RW respectively. Polymer solutions examined for rheological experiments were fresh samples. For this purpose, suitable amounts of polymer were dissolved into the specific fluid under stirring in a paddle mixer. Here, we have taken some precautions. The polymer was gradually incorporated into the mixer containing the solvent under strong mixing. After a few minutes, the stirring speed was reduced, and the solution was gently stirred overnight for complete dissolution of polymer powder. Lastly, the solution was collected and stored under vacuum.

### 2.5. FTIR Characterization

Grafting was evidenced by infrared spectroscopic analysis. The IR spectra of xanthan, polyacrylamide and xanthan-g-polyacrylamide were performed on a Thermo Scientific Nicolet IS 10 apparatus equipped with an ATR element (Smart iTR). Measurements were completed under a protocol of 40 scans and 2 cm^−1^ determination, over wavenumber extent from 4000 to 600 cm^−1^. 

### 2.6. Rheology of Polymer Solutions

Xanthan and grafted xanthan rheology analysis was done under controlled shear rate with T.A. Instruments AR-2000 rheometer. Polymer flow was examined using Couette cell geometry with a 1 mm gap between the coaxial cylinders, to prevent solvent evaporation during the tests; a trap is set up on the cell. Steady shear tests were conducted over a shear rate limit of 0.1–400 s^−1^ (this covers the shear rate values ranged between 1 and 15 s^−1^ admitted away from the wellbore of the studied reservoir). Indeed, for the flow settings of most reservoirs, the average shear rate from the injection well was normalized to 1–20 s^−1^ [26,27]. For dynamic oscillatory shear, the frequency sweep tests were done in the linear viscoelastic regime over a frequency from 0.1 to 100 rad/s at 25 °C and constant oscillating stress. Prior to frequency sweep experiments, a preliminary stress sweep test was done to fix the linear viscoelastic domain of polymer solutions. 

## 3. Results

### 3.1. Influence of Grafting Reaction Conditions

During grafting, functionalized acrylamide reacts with the backbone xanthan to form xanthan-g-polyacrylamide. The graft copolymerization can be initiated either by conventional chemical treatment or by ionizing radiation. Under microwaves, graft copolymerization appears more selective and productive, the reaction results in high grafting efficiency. With conventional methods, the competing homopolymerization lowers the grafting yields [28,29]. Here, the proposed mechanism on polymer is that the polar reactive CH_2_-OH adjacent groups on the xanthan backbone absorb the MW energy and dissociate, thus producing a macro radical onto the polymer chain. On APS initiator, the initiation mechanism is that MW heating generates dissociation of persulfate and formation of sulfate ion free radicals (SO_4_•), which on reaction with water gives •OH free radicals. Next, SO_4_•- and •OH free radicals attack the xanthan macromolecule resulting in the formation of macroradicals on the xanthan backbone. The Xan-O• macroradicals react with AAm monomer and generate XG-based AAm free radicals. The activated monomer subsequently reacts with another monomer and propagates the chain reaction, stemming graft copolymer. Alternately, the •OH free radicals may react with monomer and generate monomer free radicals. The activated monomer subsequently reacts with free radical sites of xanthan stemming graft copolymer. In this work, the microwave-assisted grafting reaction was achieved with and without initiator. As expected, from the results of Table 2 it can be observed that graft copolymerization in the presence of chemical initiator provided higher grafting parameters. 

The optimal conditions that ensure the most effective grafting parameters have been established by optimizing the reaction with respect to xanthan and acrylamide concentration, to power and time of irradiation; in this way ranges of graft copolymers were prepared. Depending on the xanthan amount and acrylamide monomer concentration, Table 3 shows that grafting parameters are increased with the increase in monomer concentration.

In contrast, from Table 4, the grafting parameters are independent of the xanthan amount.

From the results in Table 5 and Table 6, it can be observed that when the power and time of irradiation are increased, there occurs a corresponding increase in percentage grafting and grafting efficiency, whereas homopolymer formation reduces. The concentration of APS initiator was kept to 0.22 g/L for all sets of experiments. 

Lastly, for an effective graft copolymerization reaction, the optimized settings adopted are: Xanthan conc. = 0.2 g/L; AAm conc. = 8.0 mmol; Power = 750 watts; Irradiation Time = 100 s. With these settings, the best grafting parameters are obtained as following: *G* (%) = 269.00%; *GE* (%) = 94.72%; *H* (%) = 5.28%. 

### 3.2. Polymers Characterization

#### FTIR Characterization

**Figure 1:**
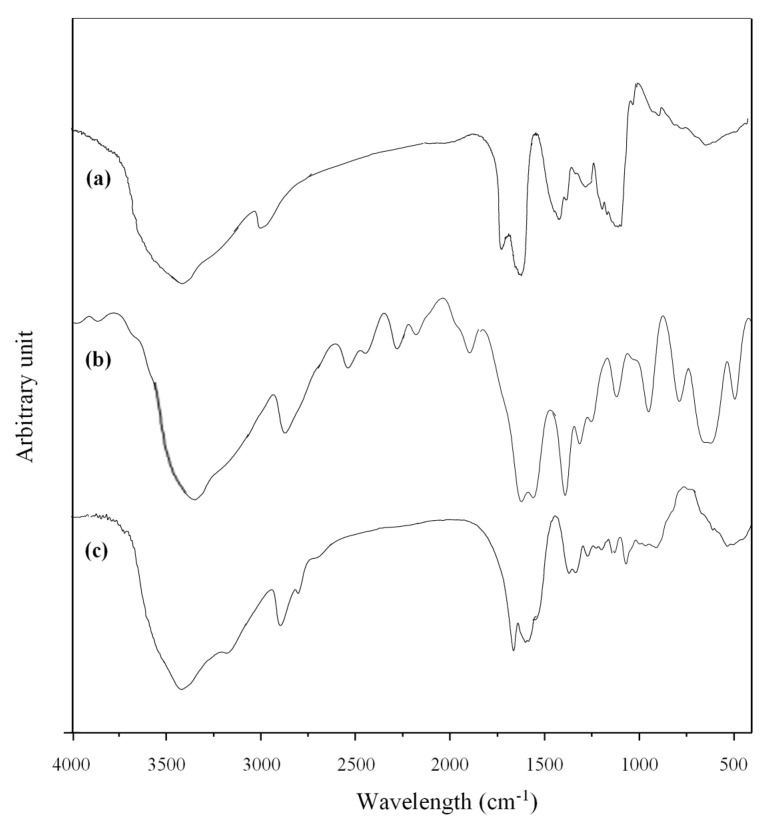
FTIR spectra of xanthan (**a**), polyacrylamide (**b**) and xanthan-g-polyacrylamide (**c**).

Polymers FTIR spectra are shown in Figure 1. 

For xanthan (Figure 1a) the spectrum displays a characteristic broad band at 3410 cm^−1^ assigned to elongation of hydroxyl group. Peaks at 1735 cm^−1^ and 1628 cm^−1^ are ascribed to the C−O vibration of alkyl esters and asymmetrical stretching of carboxylate, respectively. Further specific bands of xanthan that appear at 1410 cm^−1^ and 1065 cm^−1^ are assigned to C–H bending of methyl group and C–O stretching of alcohol, respectively. 

Polyacrylamide spectrum (Figure 1b) first presents two bands at 3375 cm^−1^ and 3185 cm^−1^ allocated to asymmetrical and symmetrical elongation of N–H bond. A specific band of C–H stretching bonds appears then at 2880 cm^−1^. Characteristic bands of first-amide (C–O elongation) and second-amide (N–H bending) are displayed at 1670 cm^−1^ and 1610 cm^−1^. The peak at 1410 cm^−1^ refers to elongation of C–N, while that at 960 cm^−1^ is related to the C–H bending out of plane. As expected, xanthan-g-polyacrylamide copolymer exhibited a combination of the absorption bands of xanthan and polyacrylamide, indicating that grafting was successful (Figure 1c). The broad band displayed at 3420 cm^−1^ is related with the overlap between the stretching bands of N–H amide group and O–H of xanthan. The band at 1675 cm^−1^ is related to superposition bands of amide-I (C–O elongation) and asymmetrical C–O stretching of carboxylate amide group. The peak at 1615 cm^−1^ is assigned to amide-II (NH bending) band of amide group. Additionally, at 1410 cm^−1^, the broad absorption band results from the overlay of the C–H bending band of xanthan methyl group and the C–N stretching band of polyacrylamide.

### 3.3. Rheological Analysis

The flow behavior of polymer solutions changes in shear viscosity under temperature and reservoir salinity and viscoelastic properties of these solutions are all sensitive parameters to describe the interface of oil/polymer solution system. As part of our main objective, polymer flooding ability of xanthan and grafted xanthan solutions has been studied in terms of their performance under various steady and oscillatory shear environments and conditions.

#### 3.3.1. Steady Shear Viscosity of Xanthan and Grafted Xanthan Solutions

**Figure 2:**
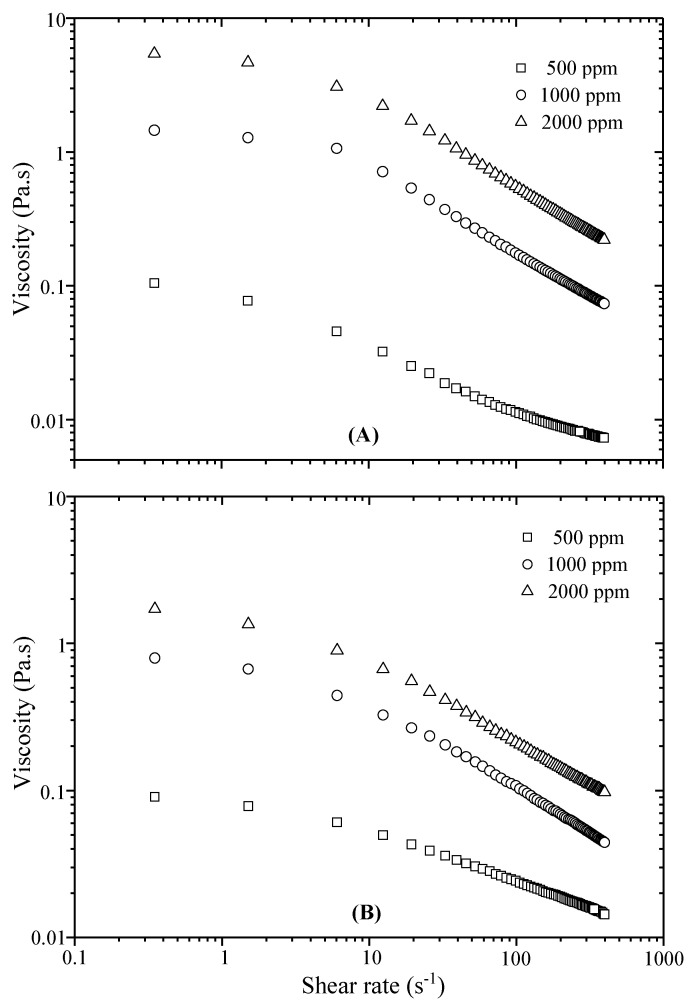
Influence of polymer concentration on shear viscosity for xanthan (**A**) and grafted xanthan (**B**)-deionized water solutions.

In the first part, evolution of shear viscosity of the prepared polymer solutions was reported in relation with polymer concentration, temperature and brine nature. In the literature, the flow behavior of xanthan was reviewed very well; it has been shown that xanthan solutions exhibited pseudoplastic behavior with shear-thinning character. Furthermore, values of apparent viscosity were most often correlated by the power law equation throughout a broad array of shear rates. The xanthan solutions offer a significant performance in salt resistance but are deficient in elasticity and their application is limited in the high temperature reservoirs. To prevail over these shortcomings, some attempts are made to improve the behavior of xanthan solutions in the high temperature reservoirs. For this purpose, the approach that was adopted in this work consists of the graft copolymerization of acrylamide onto xanthan to produce graft copolymers with improved viscoelastic properties. Experimental results of the apparent viscosity changes (Pa.s) with shear rate (s^−1^) are displayed in Figure 2A for xanthan and Figure 2B for xanthan-g-polyacrylamide, respectively. Polymer concentrations used are of 500, 1000 and 2000 ppm.

In the region of very low rates, apparent viscosity does not change much, and then begins to drop sharply. This non-Newtonian shear-thinning behavior was expected for this kind of polymer. As seen in Figure 2, at all shear rates, the apparent viscosity is directly proportional to polymer concentration; for a fixed concentration, viscosity is reduced as shear rate is increased. 

Values of initial viscosity (*η_0_*) at zero-shear rate are significantly boosted with increase in polymer concentration; this rise is particularly obvious in the low shear rate region (<10 s^−1^). At higher shear rates, the difference in viscosity values between the three concentrations becomes narrower. In the plotted curves of Figure 2, viscosity changes of polymer solutions show initially a Newtonian flow in the zone of very low shear rates; over this limited shear range, the value of *η*_0_ remains constant. Afterwards, as shear rate increases, a shear-thinning region takes place. In this zone, apparent viscosity of polymer solutions reduces according to a power law and subsequently moves to a limiting value of infinite-shear viscosity (*η_0_*). At the start of shearing, at low rates, macromolecular chains are entangled, and so in these conditions the high viscosity of solutions is due the resistance to flow. As the shear rate increases gradually, the aggregates formed by these entanglements are broken and macromolecules organize along the flow direction; at this time the flow resistance decreases and leads to a drop in viscosity. As seen from the plots, shear thinning of solutions was conserved at all polymer concentrations. At intermediate concentration (1000 ppm), both polymers show a minor viscosity loss, which can be relatively easy to recover. However, at low concentration (500 ppm), the loss is very important, and it seems that at this level the grafted xanthan with less loss behaves better than xanthan. 

**Figure 3:**
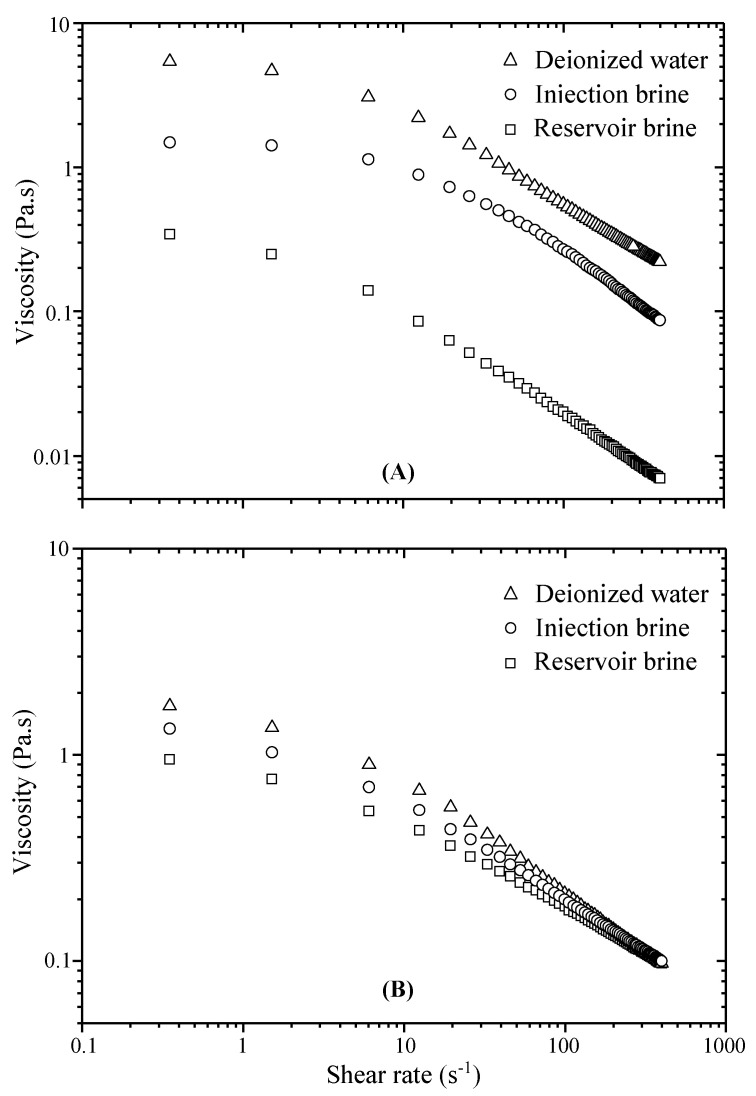
Influence of brine salinity on shear viscosity for xanthan (**A**) and grafted xanthan-brine solutions (**B**). (Polymer concentration = 2000 ppm).

Finally, it can then be said that the concentration of polymer is a key factor to increase the shear viscosity. Consequently, this increase in viscosity can adjust the poor water/oil mobility ratio responsible for poor water flood performance. Therefore, in our subsequent rheological studies the recommended concentration used for polymer solutions was fixed to 2000 ppm. Moreover, in order to improve the sweep efficiency, all EOR techniques suggest a strict control of shear viscosity of solutions at the wellbore under oilfield conditions, i.e., salinity or temperature. In many oil fields, deposit water below the hydrocarbons is usually salty; the presence of high concentrations of sodium chloride and divalent ions affects the polymer behavior during flooding. For example, at high salinity, xanthan polymer seems less sensitive to mechanical shear because of its rigid rod-like conformation. Contrastingly, synthetic polymers used in EOR (i.e., polyacrylamides) are affected by the aqueous phase salinity because of the screening effect of charged polymer chains. Accordingly, resistance to shearing of polyacrylamide solutions in brines becomes a significant parameter for their eligibility for polymer flooding. In most cases, the shear degradation under salts occurs at different levels governed by polymer type [30]. From this perspective, it will be interesting to examine the influence of injection or reservoir water on the shear viscosity of the grafted copolymer. As for xanthan, the influence of salts will be decisive on the viscosity of the grafted xanthan. In Figure 3, we report the evolution of apparent viscosity on shear rate for xanthan-brines (Figure 3A) and for grafted xanthan-brine systems (Figure 3B).

As seen in the Figures, the shear thinning of both polymer solutions is conserved in the presence of brines; moreover, viscosity losses in saline solutions are better controlled in grafted xanthan solutions. Data from viscosity-shear rate function are usually fitted by some flow equations; among those the power law equivalence covers only a limited array of shear rates; indeed, low-shear (*η_0_*) and very high-shear (*η_∞_*) ranges are poorly fitted. The Cross model (Equation (4)) covers the entire shear rate range; it was developed to clarify viscosity behavior of non-Newtonian fluids [31,32]:

(4) $$\eta_{a_{FP}}=\eta_{o}+\frac{\left(\eta_{0}-\eta_{o}\right)}{\left[1+{\left(\alpha_{c}\cdot \dot{\gamma }\right)}^{m}\right]}$$

where *η*_app_ (Pa.s) is the apparent viscosity of polymer solutions, *η_∞_* and *η_0_* (zero-shear and infinite-shear viscosities) are related to the Newtonian limits of the polymer viscosity in the low and high shear rates, and *α_c_* (s) is a constant related to the relaxation time of polymer and m is a dimensionless exponent. Fit values of Cross parameters are displayed in Table 7 for 2000-ppm polymer solutions.

As seen, polymer solutions presented a good fit to the Cross equation; m values tending to 1 indicate that the solutions were significantly shear thinning as salt concentration is decreased; compared to xanthan the grafted xanthan solutions are less shear thinning. Yield stress or zero-shear viscosity noticed for all polymer solutions at low shear rates is proportional to the concentration of polymer solutions and decreases in the presence of salts. In accordance with the nominal injection rates within our specific reservoir (10–15 s^−1^), critical value of apparent viscosity (*η_cr_*) for polymer solutions was considered at a shear rate value of 12.5 s^−1^. These values are presented in Table 8 for 2000 ppm xanthan and grafted xanthan solutions in the brines used. 

**Figure 4:**
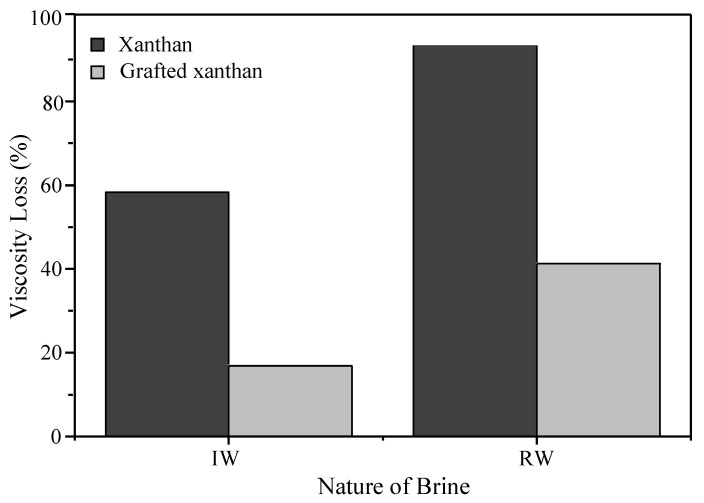
Viscosity loss ratio of polymer-brine solutions.

For grafted xanthan solutions, the values are quite close, which means that the viscosity losses due to salinity are minimal, compared to xanthan solutions. The viscosity loss due to salt degradation (*Φ_s_* %) was estimated by the following equation:

(5) $$\Phi_{s}(\%)=\frac{\eta_{DW}-\eta_{IW}}{\eta_{DW}}.100$$

where *η_DW_* (Pa.s) is the viscosity of polymer-deionized water at 12.5 s^−1^ shear rate and 25 °C; *η_IW_* (Pa.s) the viscosity of polymer-injection/reservoir water at the same settings. Figure 4 shows the viscosity losses due to salinity at nominal injection rate; thus, for xanthan, the loss reaches approximately 58.3% for solutions in injection water and 93.2% in reservoir water. However, these losses were reduced to 16.9% and 41.3%, respectively, for the grafted xanthan solutions. As an example, for the effect of polymer concentration, at the wellbore nominal injection rate the viscosity loss for xanthan solutions in deionized water was estimated at 67.8% when the polymer concentration is reduced by half. Another halving boosts dramatically the loss to 98.5%. In comparison, for xanthan-g-polyacrylamide solutions, these losses are lower: they were 51.4% and 92.6%, respectively. From this, it can then be said that polymer concentration is another key factor to control the shear viscosity.

**Figure 5:**
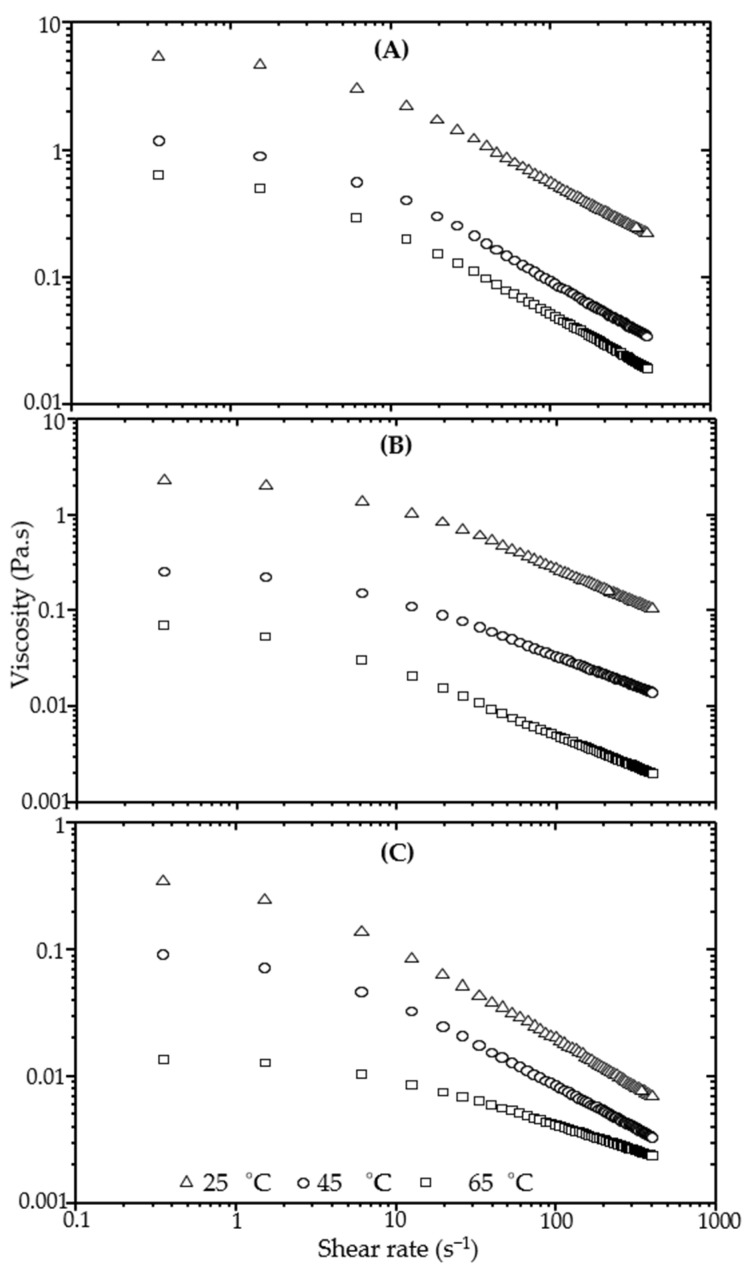
Influence of temperature on shear viscosity of xanthan-DW (**A**), xanthan-IW (**B**) and xanthan-RW solutions (**C**).

**Figure 6:**
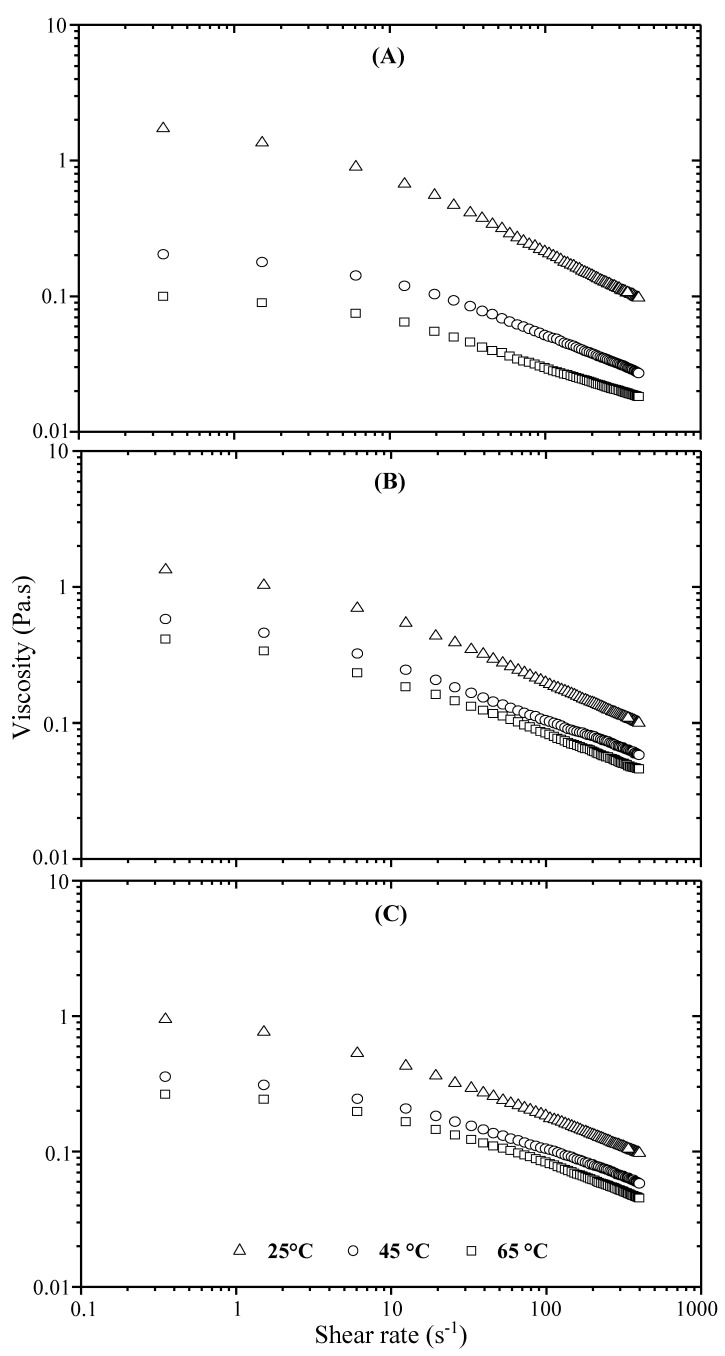
Influence of temperature on shear viscosity of grafted xanthan-DW (**A**), grafted xanthan-IW (**B**) and grafted xanthan-RW solutions (**C**).

Influence of temperature on flow behavior of 2000-ppm polymer solutions has been examined at 25, 45 and 65 °C; the latter is close to the reservoir temperature of the oil field. Figure 5 for xanthan and Figure 6 for grafted xanthan show the shear rate dependence of apparent viscosity at the three temperatures for solutions in DW (A), in IW (B) and in RW (C). All polymer solutions exhibit a drop in viscosity when the temperature is increased, and this loss is emphasized by the presence of salts. From the Figures, we can also notice that in saline solutions when the temperature is raised to 45 then to 65 °C, all the viscosity values of the grafted xanthan are higher than those of xanthan, thus suggesting that the graft copolymer is more resistant to degradation. This is more evident from Table 8, if we look at the evolution of *η_cr_* values with temperature at nominal injection rate. Therefore, at the oil well level, it seems that the degradations due to salinity and temperature generate drastic losses for xanthan viscosity; on the other hand, the viscosity of the grafted polymer appears to be better controlled.

The viscosity loss due to temperature degradation *Φ_T_* (%) was estimated by the following equation:

(6) $$\Phi_{T}(\%)=\frac{\eta_{25}-\eta_{T}}{\eta_{25}}.100$$

**Figure 7:**
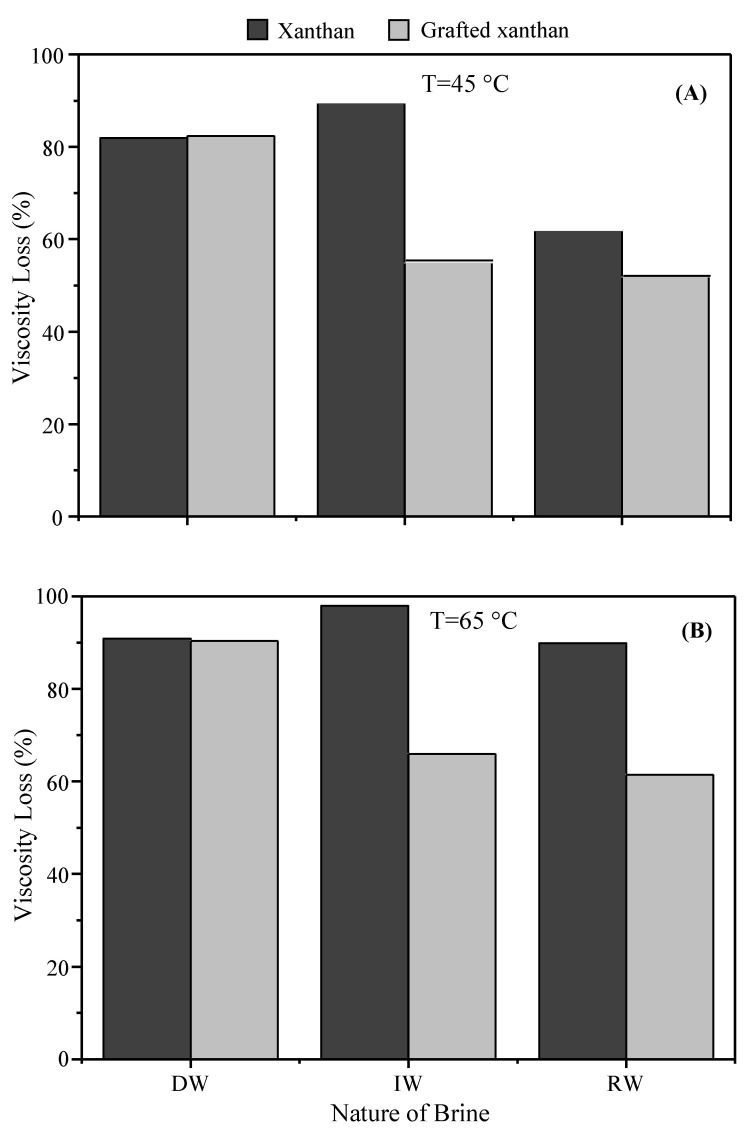
Viscosity loss ratio at 45 °C (**A**) and 65 °C (**B**) of polymer solutions.

Where *η_25_* (Pa.s) is the viscosity at shear rate of 12.5 s^−1^ and 25 °C; *η_T_* (Pa.s) the viscosity at the same shear rate and temperatures of 45 °C then 65 °C. In Figure 7, we report the losses of polymer viscosity for the temperature rise to 45 °C (Figure 7A), and further to 65 °C (Figure 7B). 

As seen for solutions in deionized water, the viscosity losses due to temperature are extremely high and the same losses are observed for both polymers. Thus, the polymer solutions retain about 18% of their initial viscosity when the temperature is raised to 45 °C. At around reservoir temperature (65 °C), viscosity loss reaches more than 90%. For solutions in injection water the degradation due to temperature is more critical for xanthan solution; thus, for the first temperature rise to 45 °C, xanthan holds only 10% of its initial viscosity, while at the same time the grafted xanthan solution keeps up more than 45% of *η_25_*. At 65 °C, viscosity of the xanthan solution is almost completely lost while the grafted xanthan still preserves more than 35%. Surprisingly, in the brinier reservoir water, the viscosity losses of both polymer solutions are more controlled; this applies especially to the grafted polymer.

#### 3.3.2. Oscillatory Shear of Grafted Xanthan Solutions

Besides the shear viscosity, viscoelasticity of the polymer solutions is a significant factor for the choice of suitable polymer for the EOR projects. Usually, highly elastic fluids exhibit notably high-level pressure drop during flow through porous media [33]. Therefore, controlling viscoelastic behavior of injected fluids is an additional key factor in modifying oil-displacing efficiency. Indeed, owing to a suitable elasticity these solutions can drive the remaining oil kept in the porous media [34,35]. Xia et al. observed that polymer solutions with high elastic characteristics could displace residual oil of various natures even without improving the pressure gradient [36]. 

**Figure 8:**
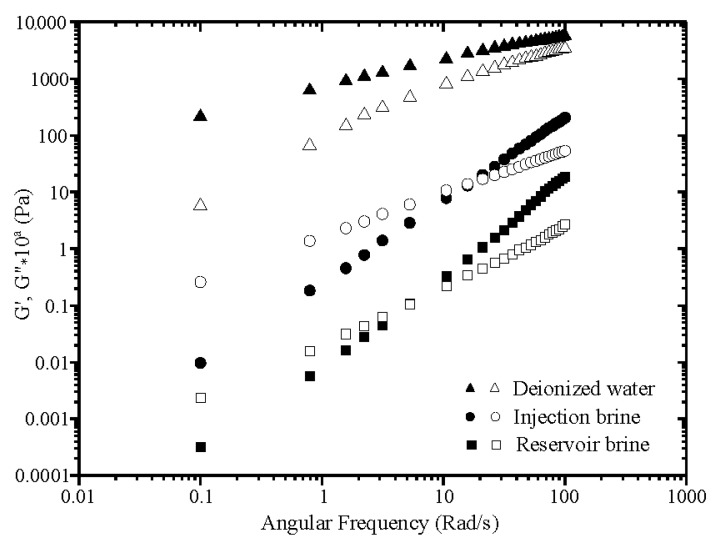
Mechanical spectra of xanthan solutions in DW, IW and RW (filled symbol G’, open symbol G”). (To avoid overlapping, data have been vertically shifted by 10^a^ with a = 0, 1, 2).

**Figure 9:**
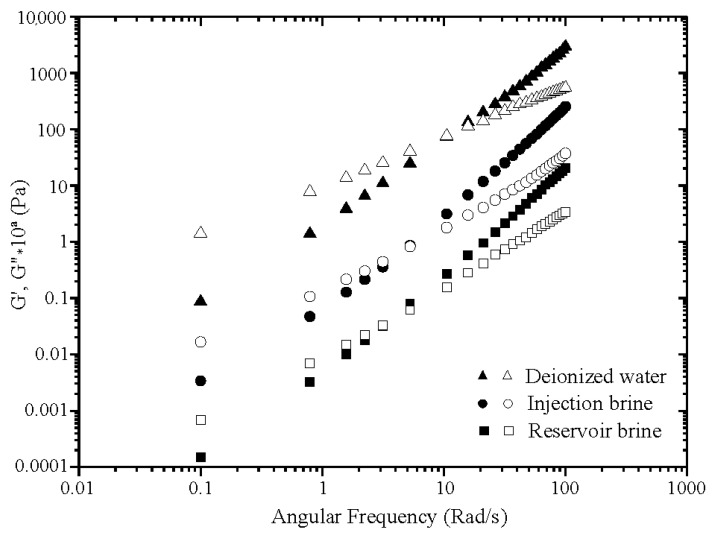
Mechanical spectra of grafted xanthan solutions in DW, IW and RW.

In the second part of rheological analysis, oscillatory tests have been performed to clarify the dynamic performance of polymer solutions in the linear domain. Oscillatory experiments were carried out with constant stress in the linear limits as defined from the initial stress sweep curves. Viscoelastic properties of polymer solutions are expressed by the elastic and viscous moduli (Pa). The former (G’) measures the reversible saved energy of the system while the latter (G”) quantifies the energy loss. The viscoelastic characteristics of xanthan solutions in dynamic regime are well known; several works have clarified the behavior of this polysaccharide under oscillatory shear [37]. To our knowledge, dynamic rheology of xanthan-g-polyacrylamide has not yet been studied; an attempt is made here to clarify dynamic behavior of the grafted polymer solutions. Frequency sweeps were conducted for 2000 ppm polymer solutions at 25 °C and over 0.1–100 Rad/s of frequency. Figure 8 for xanthan and Figure 9 for grafted xanthan display evolution of G’ and G” according to frequency (ω) for solutions in DW, IW and RW. To avoid overlapping of the curves, data were vertically shifted by a factor of 10^a^ (with a = 0, 1, 2…). From Figure 8, the xanthan solution in DW shows behavior of semi-dilute “weak gels”. In this state, the individual polymer chains interact, overlap and adopt an expanded coil conformation as expected. 

The mechanical spectrum shows that the elastic modulus G’ remains well above G” over all the applied frequencies, with a weak dependence on frequency for both moduli. An additional dynamic characteristic, the viscoelastic ratio or phase angle (δ) tangent (tan δ = G”/G’) varies from 0.6 to 0.03 in low frequencies, indicating that the elastic nature prevailed over the viscous one. In the brines, IW and RW, xanthan solutions behave as entangled viscoelastic polymers; G’ and G” become more dependent on frequency in the form of G’~ω^n’^ and G”~ω^n”^ with n’ and n”, slopes of the power dependence. At low frequencies (terminal zone) G’’ exceeds G’, therefore G’ crosses G”, afterward with increasing frequency, value of G’ becomes larger than that of G”. The polymer solutions become gradually more elastic with an increase in salinity, as evidenced by the move of the crossover between G’ and G” to lower frequencies. The shift from the viscous to the prevalent elastic character occurs at a frequency of 15.79 rad/s for polymer solutions in IW and 5.27 rad/s for solutions in RW. The drop of crossover frequency in the presence of salts has already been observed for xanthan by Rochefort [38]. In contrast with solutions in DW, tan δ varies inversely in IW and RW with low values at high frequencies (0.26 and 0.14, respectively). In Figure 9, the mechanical spectra show that xanthan-g-polyacrylamide solutions behave as semi-dilute entangled systems in the three media and no “weak gel” was formed. The same behavior of that of xanthan in saline solutions is observed: for all polymer solutions G’ and G” are frequency dependent. From the values of power law exponents, the frequency dependences of both moduli are of the form G’~ω^2^ and G”~ω. This confirms the behavior of grafted xanthan solutions as fluid-like entanglement networks; at very low frequencies (in the terminal zone), they flow as highly viscous liquids. 

For all sample solutions, the values of the parameters n’ and n” of frequency power dependence of G’ and G” are summarized in Table 9. 

Compared to xanthan, the elastic properties of xanthan-g-polyacrylamide have been significantly improved in saline media; this enhancement is obviously due to the contribution of the polyacrylamide’s chain flexibility. The shift from the viscous to the prevalent elastic character occurs at a crossover frequency of 10.53 rad/s for polymer solutions in deionized water, 5.27 rad/s in IW and 3.15 rad/s for solutions in RW.

## 4. Conclusions

As a potential system to regulate a suitable value of the viscosity needed for drilling fluid applications, xanthan-g-polyacrylamide was synthesized using fast microwave-assisted grafting. The graft copolymer offers an important opportunity to be used for polymer flooding in a specific oil field of geological Devonian nature. For this handling, the grafted xanthan solutions should withstand the particular well nominal injection rates (10–15 s^−1^), temperature (~68 °C) and salinity of the reservoir water. In search of the optimal conditions that ensure the most effective grafting parameters to produce such a copolymer, the grafting reaction was optimized in terms of reactants’ concentration, irradiation power and reaction time. Evidence of grafting has been physico-chemically confirmed by infrared spectroscopy (FTIR). Subsequently, the rheological characteristics of xanthan and grafted xanthan solutions have been studied by varying some key parameters such as concentration, salinity and temperature; results were treated and related with viscosity and elasticity. In all cases, the steady shear viscosity of the polymer solutions exhibited pseudoplastic flow and shear-thinning behavior. 

In addition, the viscosity losses of grafted xanthan solutions are more controlled and large parts of these losses due to salinity or temperature have been mostly absorbed. From dynamic measurements, it is demonstrated that the elastic properties of xanthan-g-polyacrylamide have been notably upgraded compared to xanthan. Otherwise, the grafted samples are more stable in the presence of salts and exhibit more elasticity as salinity increases. Therefore, both flow and dynamic experimental results are in good agreement for analyzing the behavior of the grafted xanthan solutions according to polymer concentration, temperature and salinity. Graft-copolymerization of acrylamide onto xanthan gives rise to attractive rheological behaviors in temperature and especially in briny environments. Graft polymer solutions with increased viscosity and elasticity can better control the suitable mobility of the oil/polymer system, which consequently improves the sweep efficiency of the transferring solution. From our perspective, the rheological analysis of xanthan-g-polyacrylamide solutions showed that the prediction for polymer flooding in the particular Devonian oilfield could technically be successful.

## Figures and Tables

**Table 1:** Chemical composition of brines used for rheology tests.

Parameters	Nature of Brine Water	
	Reservoir Formation Water	Injection Water
pH	7.5	4.3
Composition (g/L)		
Ca^2+^	0.24	0.27
Mg^2+^	0.17	0.06
Na^+^	0.23	0.76
K^+^	0.013	0.04
Sr^2+^	0.0007	0.003
Cl^−^	1.04	0.56
HCO_3_^−^	0.21	0.20
SO_4_^2−^	0.11	1.30
Salinity (g/L)	1.71	0.92
Turbidity (NTU)	79.8	2.00
Dry extract (g/L)	3.24	4.10

**Table 2:** Effect of APS initiator on grafting parameters (power = 750 Watt; t = 100 s; mXan = 0.2 g/L; mAAm = 8.0 mmol).

Grafting Method	*G* (%)	*GE* (%)	*H* (%)
In the presence of APS	269.00	94.72	5.28
In the absence of APS	228.50	80.45	19.55

**Table 3:** Effect of monomer concentration on grafting parameters (power = 750 Watt; t = 100 s; mxan = 0.2 g/L).

AAm Concentration (mmol)	*G* (%)	*GE* (%)	*H* (%)
2.50	48.00	57.14	42.86
3.80	81.00	60.44	39.55
5.20	200.50	85.68	14.32
6.60	203.50	86.96	13.03
8.00	269.00	94.72	5.28
10.80	235.00	87.23	12.76

**Table 4:** Effect of xanthan amount on grafting parameters (Power = 750 Watt; t = 100 s.; mAAm = 8.0 mmol).

Xanthan Amount (g/L)	*G* (%)	*GE* (%)	*H* (%)
0.10	505.00	88.90	11.10
0.15	356.66	94.19	5.81
0.20	269.00	94.71	5.28
0.25	191.20	75.35	24.65
0.30	135.33	71.47	28.53
0.35	62.28	38.38	61.62

**Table 5:** Effect of irradiation power on grafting parameters (t = 100 s.; mxan = 0.2 g/L; mAAm = 8.0 mmol).

Power (Watt)	*G* (%)	*GE* (%)	*H* (%)
150	101.00	35.56	64.43
350	116.50	41.02	59.00
500	166.50	58.62	41.38
650	245.50	86.44	13.56
750	269.00	94.72	5.28

**Table 6:** Effect of irradiation time on grafting parameters (Power = 750 Watt; mxan = 0.2 g/L; mAAm = 8.0 mmol).

Time (s)	*G* (%)	*GE* (%)	*H* (%)
30	130.50	45.95	54.05
40	180.50	63.55	36.45
50	228.50	80.45	19.55
100	269.00	94.72	5.28
150	281.50	99.11	0.88
200	277.50	97.71	2.29

**Table 7:** Cross coefficients for 2000 ppm polymer solutions (T = 25 °C).

Polymer	*η_0_* (Pa.s)	*η_∞_* (Pa.s)	*α_c_* (s)	*m*	R^2^
Polymer-deionized water					
Xanthan	5.85 ± 14.8 x 10^−3^	0.077 ± 3.8 × 10^−3^	0.146	0.89 ± 4.7 × 10^−3^	0.9999
Grafted xanthan	2.01 ± 13.3 × 10^−3^	0.010 ± 2.8 × 10^−3^	0.227	0.69 ± 7.1 × 10^−3^	0.9997
Polymer-Injection water					
Xanthan	2.44 ± 12.3 × 10^−3^	0.019 ± 4.0 × 10^−3^	0.116	0.86 ± 9.5 × 10^−3^	0.9996
Grafted xanthan	1.67 ± 11.1 × 10^−3^	0.012 ± 2.1 × 10^−3^	0.297	0.60 ± 5.4 × 10^−3^	0.9998
Polymer-Reservoir water					
Xanthan	0.40 ± 2.2 × 10^−3^	0.0014 ± 0.25 × 10^−3^	0.375	0.84 ± 6.4 × 10^−3^	0.9998
Grafted xanthan	1.18 ± 5.8 × 10^−3^	0.012 ± 1.5 × 10^−3^	0.239	0.56 ± 3.9 × 10^−3^	0.9999

**Table 8:** Viscosity dependence of 2000 ppm polymer solutions at 12.5 s^−1^ shear rate.

Temperature (°C)	Viscosity at 12.5 s^−1^ Shear Rate and 2000 ppm Polymer Concentration (Pa.s)		
	Deionized Water	Injection Brine	Reservoir Brine
25	0.671	0.540	0.430
45	0.119	0.245	0.208
65	0.065	0.184	0.166

**Table 9:** Values of the power dependence of G’ and G” moduli.

Polymer	G’		G”	
	n’	R2	n”	R2
Polymer-deionized water				
Xanthan	0.4 ± 0.008	0.9960	0.6 ± 0.01	0.9968
Grafted xanthan	1.8 ± 0.024	0.9988	0.8 ± 0.01	0.9992
Polymer-Injection water				
Xanthan	1.4 ± 0.008	0.9998	0.7 ± 0.003	0.9999
Grafted xanthan	2.0 ± 0.012	0.9997	1.4 ± 0.023	0.9984
Polymer-Reservoir water				
Xanthan	1.8 ± 0.03	0.9982	1.2 ± 0.02	0.9981
Grafted xanthan	1.9 ± 0.02	0.9995	1.3 ± 0.01	0.9997

## Data Availability

The data presented in this study are available on request from the corresponding author.
